# Radiomic markers of intracerebral hemorrhage expansion on non-contrast CT: independent validation and comparison with visual markers

**DOI:** 10.3389/fnins.2023.1225342

**Published:** 2023-08-16

**Authors:** Stefan P. Haider, Adnan I. Qureshi, Abhi Jain, Hishan Tharmaseelan, Elisa R. Berson, Tal Zeevi, David J. Werring, Moritz Gross, Adrian Mak, Ajay Malhotra, Lauren H. Sansing, Guido J. Falcone, Kevin N. Sheth, Seyedmehdi Payabvash

**Affiliations:** ^1^Department of Radiology and Biomedical Imaging, Yale School of Medicine, New Haven, CT, United States; ^2^Department of Otorhinolaryngology, University Hospital of Ludwig-Maximilians-Universität München, Munich, Germany; ^3^Zeenat Qureshi Stroke Institute and Department of Neurology, University of Missouri, Columbia, MO, United States; ^4^Stroke Research Centre, University College London, Queen Square Institute of Neurology, London, United Kingdom; ^5^Department of Neurology, Yale School of Medicine, New Haven, CT, United States

**Keywords:** cerebral hemorrhage, hematoma, machine learning, computed tomography, radiomics

## Abstract

**Objective:**

To devise and validate radiomic signatures of impending hematoma expansion (HE) based on admission non-contrast head computed tomography (CT) of patients with intracerebral hemorrhage (ICH).

**Methods:**

Utilizing a large multicentric clinical trial dataset of hypertensive patients with spontaneous supratentorial ICH, we developed signatures predictive of HE in a discovery cohort (*n* = 449) and confirmed their performance in an independent validation cohort (*n* = 448). In addition to *n* = 1,130 radiomic features, *n* = 6 clinical variables associated with HE, *n* = 8 previously defined visual markers of HE, the BAT score, and combinations thereof served as candidate variable sets for signatures. The area under the receiver operating characteristic curve (AUC) quantified signatures’ performance.

**Results:**

A signature combining select radiomic features and clinical variables attained the highest AUC (95% confidence interval) of 0.67 (0.61–0.72) and 0.64 (0.59–0.70) in the discovery and independent validation cohort, respectively, significantly outperforming the clinical (*p*_discovery_ = 0.02, *p*_validation_ = 0.01) and visual signature (*p*_discovery_ = 0.03, *p*_validation_ = 0.01) as well as the BAT score (*p*_discovery_ < 0.001, *p*_validation_ < 0.001). Adding visual markers to radiomic features failed to improve prediction performance. All signatures were significantly (*p* < 0.001) correlated with functional outcome at 3-months, underlining their prognostic relevance.

**Conclusion:**

Radiomic features of ICH on admission non-contrast head CT can predict impending HE with stable generalizability; and combining radiomic with clinical predictors yielded the highest predictive value. By enabling selective anti-expansion treatment of patients at elevated risk of HE in future clinical trials, the proposed markers may increase therapeutic efficacy, and ultimately improve outcomes.

## Introduction

In patients with acute spontaneous intracerebral hemorrhages (ICH), growth of the hematoma volume after hospital admission (“hematoma expansion,” HE) is associated with early clinical deterioration, worse long-term functional outcome, and higher mortality (Brott et al., 1997; Davis et al., 2006; Lord et al., 2015; Hostettler et al., 2019). In the absence of established effective treatments for ICH patients, HE represents a potential therapeutic target (Tanaka and Toyoda, 2021). Identification of patients at elevated risk of HE by means of (imaging) biomarkers or risk scores may allow selective treatment of individuals who likely benefit from anti-expansion therapies in future trials.

In addition to clinical variables (Al-Shahi Salman et al., 2018), the spot sign on admission computed tomography (CT)-angiography (CT-A) has been proposed as a predictor of HE (Demchuk et al., 2012). However, not all centers perform baseline CT-A immediately after identifying an ICH on non-contrast CT, which is the standard-of-care imaging technique for detection of intracranial hemorrhage. Moreover, CT-A is associated with additional ionizing radiation and contrast administration. Alternatively, studies suggested visual markers on non-contrast CT as predictors of ICH expansion (Boulouis et al., 2017; Morotti et al., 2018, 2019). However, overlapping definitions and subjective interpretations of imaging findings limit the applicability and generalizability of such visual markers (Morotti et al., 2019). To date, the clinical value of the CT-A spot sign and visual non-contrast CT markers remains unclear (Hostettler et al., 2019).

A possible alternative is a radiomic biomarker, which allows utilization of standard-of-care non-contrast CTs to provide an objective and reproducible characterization of hematomas (Gillies et al., 2016; Haider et al., 2020a). Radiomic analysis enables a comprehensive, quantitative assessment of shape, density, and texture attributes of volumes-of-interest in medical images through extraction of high-dimensional sets of features (Gillies et al., 2016; Haider et al., 2020a). While the focus of radiomics research thus far were oncological applications (Gillies et al., 2016; Haider et al., 2020a,b,c,d; Tomaszewski and Gillies, 2021), lately stroke radiomics has gained traction (Chen et al., 2021b; Haider et al., 2021; Avery et al., 2022). Recent studies applied radiomic analysis of baseline non-contrast CTs to predict HE; however, with some using small sample sizes, they report a wide range of prediction accuracies (Shen et al., 2018; Xie et al., 2020; Xu et al., 2020; Chen et al., 2021a,c; Pszczolkowski et al., 2021).

Given the need for generalizable imaging biomarkers of HE, which may guide therapeutic interventions in anti-expansion trials, and the equivocal predictive performance of prior radiomic models, we aimed to generate robust non-contrast CT radiomic signatures for HE prediction. Using a large, multicentric dataset of patients prospectively enrolled in the ATACH-2 (Antihypertensive Treatment of Acute Cerebral Hemorrhage II) trial, we devised and independently validated radiomic signatures predictive of ICH expansion. Then, we compared their performance with signatures consisting of visual markers of HE, clinical variables, and combined signatures.

## Materials and methods

### Data acquisition

**FIGURE 1:**
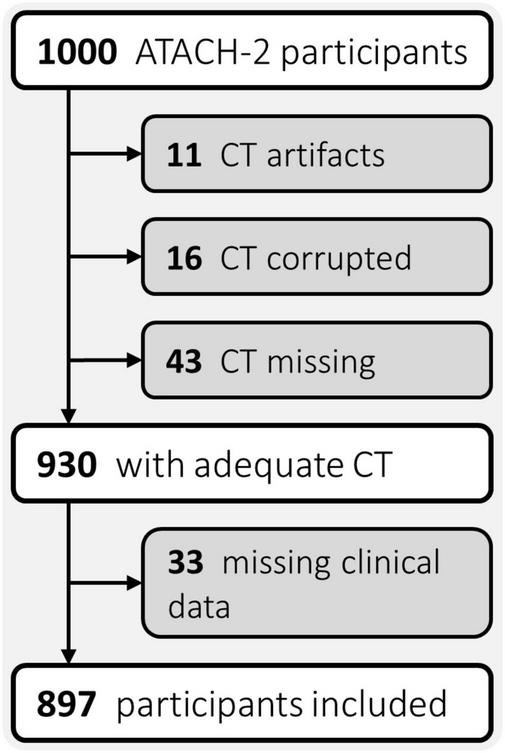
Flowchart of patient exclusion criteria. ATACH-2, Antihypertensive Treatment of Acute Cerebral Hemorrhage II; CT, computed tomography.

All clinical data and CT scans utilized in this study were gathered by the multicentric, randomized, two-group ATACH-2 trial (*n* = 1,000), which evaluated earlier and more aggressive antihypertensive treatment in patients with acute, spontaneous, supratentorial ICH, and found no significant treatment benefit (ClinicalTrials.gov identifier: NCT01176565) (Qureshi et al., 2016). Ethical compliance was ensured by the ATACH-2 investigators (Qureshi et al., 2016); our group performed *post hoc* analyses of anonymized data. For this study, trial participants with missing or corrupted baseline CT scans, severe CT artifacts affecting the ICH or missing data were excluded (Figure 1). The remainder was randomly allocated, in equal parts, to a discovery and an independent validation cohort.

### Segmentation of ICH

**FIGURE 2:**
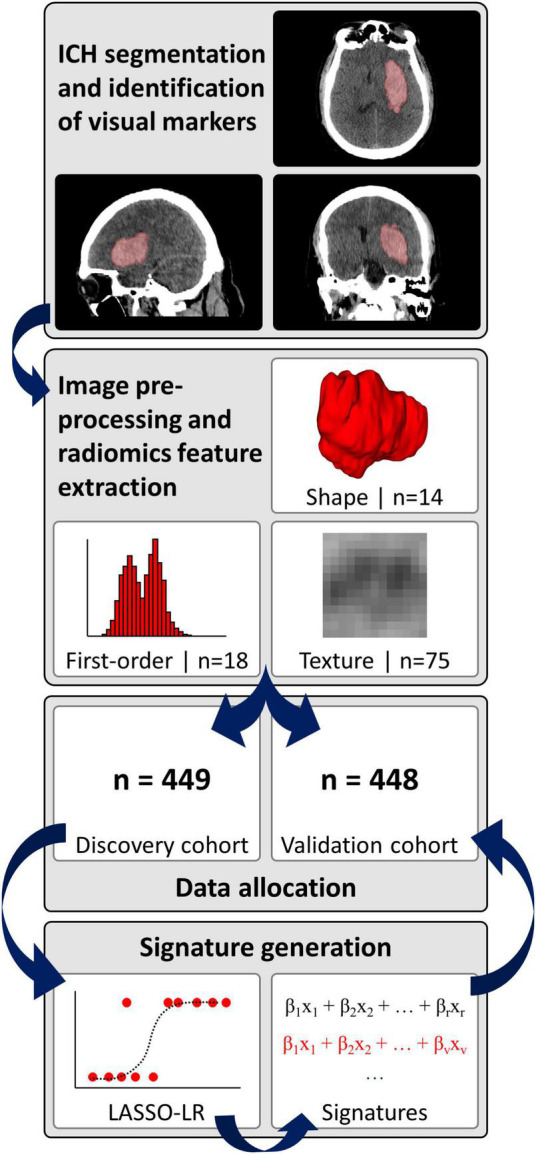
Analysis pipeline. ICH, intracerebral hemorrhage; LASSO-LR, least absolute shrinkage and selection operator-regularized logistic regression.

The baseline non-contrast head CT scans were loaded in 3D-Slicer version 4.10.1 software and the ICH contours were manually delineated slice-by-slice on axial slices (Fedorov et al., 2012), to generate three-dimensional ICH masks, as reported previously (Haider et al., 2021). Subsequently, a neuroradiologist (SP) with > 9 years of dedicated experience reviewed and adjusted all segmentations. Figure 2 summarizes the analysis pipeline from ICH segmentation to generation and final validation of signatures.

### Radiomics pipeline

We pre-processed the non-contrast CT images and corresponding hemorrhage masks and extracted radiomics information via a fully automated pipeline, as detailed in the Supplementary methods (van Griethuysen et al., 2017; Pyradiomics community, 2018; Haider et al., 2021). In brief, pre-processing included voxel dimension resampling to an isotropic 1 mm × 1 mm × 1 mm spacing using B-spline interpolation, re-segmentation of hemorrhage masks to a 1–200 Hounsfield unit density range, and generation of derivative images by applying a “coif-1” wavelet transform (*n* = 8 derivative images from applying high- and low-pass filtering in each spatial direction) as well as three Laplacian of Gaussian filters with sigma-settings of 2, 4 and 6 mm (van Griethuysen et al., 2017; Pyradiomics community, 2018; Haider et al., 2021). Finally, *n* = 14 shape, *n* = 18 first-order and *n* = 75 texture features were extracted from the original images and eleven derivative images per original, resulting in a total of *n* = 1,130 features per ICH (Supplementary Table 1).

### Visual CT markers of HE

Applying diagnostic criteria published by Morotti et al. (2019), three readers, who were blinded to each other’s reads, identified eight visual ICH markers on baseline non-contrast head CTs, including density (“blend sign,” “hypodensity,” “swirl sign,” “black hole sign,” “fluid level”) and shape markers (“island sign,” “satellite sign,” “irregular shape”) (Haider et al., 2021). Supplementary Table 2 summarizes the diagnostic criteria proposed by Morotti et al. (2019). Binary variables (i.e., visual marker present or absent) were obtained for all subsequent analyses by majority vote of the three reads.

### Signatures of HE

Hematoma expansion (HE) was defined as a binary variable by an increase in ICH volume of > 33% or > 6 ml from baseline to 24-h follow-up non-contrast head CT (Dowlatshahi et al., 2011). Using the discovery cohort, we devised weighted linear combinations of variables (termed “signatures”) to predict HE. These were generated by fitting least absolute shrinkage and selection operator-regularized logistic regression (LASSO-LR) models to the discovery cohort with HE as the dependent variable and with different sets of independent (“candidate”) variables, as detailed in the Supplementary methods. The independent validation cohort served to test the predictive performance of signatures.

To explore potential performance enhancements, we devised an iteration of our pipeline incorporating radiomic feature harmonization to correct for CT slice thickness variability prior to signature generation, as detailed in the Supplementary methods (Orlhac et al., 2022; Fortin, 2023). In brief, we applied ComBat harmonization for each radiomic feature with slice thickness as the batching variable (Orlhac et al., 2022; Fortin, 2023). To preclude data leakage, ComBat parameters were estimated from the discovery cohort only.

The “radiomics signature” was generated by supplying radiomics features to a LASSO-LR model as candidate variables. We excluded radiomics features with inadequate stability to inter- and intra-rater segmentation variability (*n* = 1,002/1,130 features retained) and high inter-feature collinearity (*n* = 429/1,002 features retained) prior to LASSO-LR fitting as detailed in the Supplementary methods (Haider et al., 2021).

The “visual signature” was generated by supplying *n* = 8 visual markers of HE to a LASSO-LR model as candidate variables. The “clinical signature” was generated by supplying clinical variables to a LASSO-LR model which exhibited significant association with HE in a large meta-analysis by Al-Shahi Salman et al. (2018), i.e., sex, baseline National Institutes of Health Stroke Scale (NIHSS) score, Glasgow Coma Scale score, platelet count, and blood glucose level. We compared the signatures to the “BAT score,” which is designed to predict HE by combining visual markers (blend sign: 1 point; hypodensity: 2 points) with the time from symptom onset to CT (< 2.5 h: 2 points) (Morotti et al., 2018).

Combined signatures were generated by supplying robust and non-collinear radiomics features (*n* = 429) along with *n* = 8 visual markers (“radiomics + visual signature”), *n* = 5 clinical variables (“radiomics + clinical signature”), the BAT score (“radiomics + BAT signature”), or all visual and clinical variables (“radiomics + visual + clinical signature”) to a LASSO-LR model.

Given the large number of radiomic features, we generated versions of the combined signatures where only radiomics features included in the radiomics signature were supplied to LASSO-LR models as candidate variables, thereby mitigating any dimensionality-related bias in LASSO-based variable selection (“select radiomics + visual signature,” “select radiomics + clinical signature,” “select radiomics + visual + clinical signature”).

The time from symptom onset to the baseline CT was additionally included in all candidate variable sets (except those sets including the BAT score) in order to scale signature scores to time post symptom onset. Continuous and ordinal candidate variables were standardized prior to analysis by subtracting the discovery cohort mean and dividing by the corresponding standard deviation (SD) per feature. We imputed the median value for missing values in clinical signature variables.

### Statistical analysis

Continuous variables are presented as means (SD) or medians (interquartile range, IQR), while categorical variables are presented as counts and percentages. *p*-values < 0.05 ascertained statistical significance. All analyses were performed in R version 3.6.0 (R Development Core Team, 2019). We calculated the area under the receiver operating characteristic curve (AUC, 95% confidence interval, CI), precision, recall, negative predictive value and F1-score to quantify the predictive performance of signatures. DeLong’s method was employed to compare AUCs and derive 95% CIs (DeLong et al., 1988). The “pROC” version 1.15.0 package for R provided all functionality for AUC-related analyses (Robin et al., 2011). We calculated Spearman’s rho to determine the association of signature scores with long-term functional outcome assessed by the modified Rankin Scale (mRS) at 90 days after randomization.

## Results

### Patients

Of the *n* = 897 patients with adequate CTs and complete clinical phenotypes (Figure 1), we randomly allocated *n* = 449 to the discovery, and *n* = 448 to the independent validation cohort. Table 1 summarizes the demographics, risk profiles, imaging characteristics, treatment, and clinical outcomes of the two cohorts as well as the presence of visual markers of HE. In the discovery and validation cohorts, *n* = 118/449 (∼26%) and *n* = 126/448 (∼28%) patients experienced HE, respectively.

**TABLE 1:** Patients’ characteristics.

	Discovery cohort	Independent validation cohort	*p*-value discovery vs. independent
Number of patients	449	448	
Male sex−*n* (%)	266 (59.2%)	282 (62.9%)	0.26
Age [years]−mean (SD)	61.9 (13.2)	62.4 (13.0)	0.68
**Race−*n* (%)**			
Asian	252 (56.1%)	260 (58.0%)	0.90
White	127 (28.3%)	127 (28.3%)	
Black or African American	62 (13.8%)	52 (11.6%)	
American Indian or Alaska Native	1 (0.2%)	1 (0.2%)	
Other or unknown	7 (1.6%)	8 (1.8%)	
**Ethnic group−*n* (%)**			
Hispanic or Latino	34 (7.6%)	35 (7.8%)	0.89
Not Hispanic or Latino or unknown	415 (92.4%)	413 (92.2%)	
**History of hypertension−*n* (%)**			
Yes	359 (80.0%)	352 (78.6%)	0.77
No	80 (17.8%)	83 (18.5%)	
Unknown	10 (2.2%)	13 (2.9%)	
**History of diabetes mellitus type I/II−*n* (%)**			
Yes	91 (20.3%)	84 (18.8%)	0.42
No	352 (78.4%)	353 (78.8%)	
Unknown	6 (1.3%)	11 (2.5%)	
**History of hyperlipidemia−*n* (%)**			
Yes	109 (24.3%)	115 (25.7%)	0.89
No	314 (69.9%)	308 (68.8%)	
Unknown	26 (5.8%)	25 (5.6%)	
**History of congestive heart failure−*n* (%)**			
Yes	11 (2.4%)	18 (4.0%)	0.40
No	433 (96.4%)	426 (95.1%)	
Unknown	5 (1.1%)	4 (0.9%)	
**History of atrial fibrillation−*n* (%)**			
Yes	11 (2.4%)	20 (4.5%)	0.24
No	434 (96.7%)	423 (94.4%)	
Unknown	4 (0.9%)	5 (1.1%)	
**History of prior stroke or TIA−*n* (%)**			
Yes	79 (17.6%)	70 (15.6%)	0.53
No	368 (82.0%)	374 (83.5%)	
Unknown	2 (0.4%)	4 (0.9%)	
**History of cigarette smoking−*n* (%)**			
Current	105 (23.4%)	125 (27.9%)	0.25
Former	84 (18.7%)	78 (17.4%)	
Never	228 (50.8%)	205 (45.8%)	
Unknown	32 (7.1%)	40 (8.9%)	
**GCS score at baseline−*n* (%)**			
3–8	12 (2.7%)	16 (3.6%)	0.59
9–11	56 (12.5%)	45 (10.0%)	
12–14	126 (28.1%)	127 (28.3%)	
15	255 (56.8%)	260 (58.0%)	
**NIHSS score at baseline−*n* (%)**			
0–4	74 (16.5%)	69 (15.4%)	0.29
5–9	130 (29.0%)	108 (24.1%)	
10–14	112 (24.9%)	129 (28.8%)	
15–19	74 (16.5%)	89 (19.9%)	
20–25	39 (8.7%)	40 (8.9%)	
> 25	17 (3.8%)	11 (2.5%)	
Unknown	3 (0.7%)	2 (0.4%)	

Blood glucose at baseline [mg/dL]−mean (SD)	140.4 (59.8)	137.4 (50.8)	0.80
Platelet count at baseline [x 10^3^/mm^3^]−mean (SD)	223.0 (62.3)	219.3 (60.1)	0.49
**Location of hematoma−*n* (%)**			
Thalamus	175 (39.0%)	172 (38.4%)	0.79
Basal ganglia	224 (49.9%)	223 (49.8%)	
Cerebral lobe	50 (11.1%)	52 (11.6%)	
Cerebellum	0 (0%)	1 (0.2%)	

Intracerebral hematoma volume at baseline [cm^3^]−mean (SD)	12.6 (12.7)	12.6 (11.2)	0.41
Intracerebral hematoma volume at 24-h follow-up [cm^3^]−mean (SD)	15.5 (17.8)	15.5 (15.4)	0.55
Intraventricular hemorrhage present at baseline−*n* (%)	132 (29.4%)	118 (26.3%)	0.31
Experienced hematoma expansion−*n* (%)^a^	118 (26.3%)	126 (28.1%)	0.53
Symptom onset to baseline CT [minutes]−mean (SD)	98.1 (49.6)	98.8 (53.0)	0.89
**CT−mean (SD)^b^**			
Slice thickness [mm]	5.2 (1.8)	5.3 (1.7)	0.06
In-plane pixel spacing [mm]	0.46 (0.03)	0.46 (0.03)	0.11
In-plane image matrix [*n* × *n*]	512 × 512	512 × 512	
**Visual CT markers of hematoma expansion−*n* (%)^c^**			
Blend sign present	39 (8.7%)	32 (7.1%)	0.39
Hypodensity present	339 (75.5%)	354 (79.0%)	0.21
Swirl sign present	31 (6.9%)	33 (7.4%)	0.79
Black hole sign present	39 (8.7%)	40 (8.9%)	0.90
Island sign present	18 (4.0%)	25 (5.6%)	0.27
Satellite sign present	65 (14.5%)	54 (12.1%)	0.28
Fluid level present	0 (0%)	2 (0.4%)	0.16
Irregular shape present	130 (29.0%)	113 (25.2%)	0.21
**BAT score (Morotti et al., 2018)−*n* (%)**			
0	21 (4.7%)	23 (5.1%)	0.39
1	2 (0.4%)	1 (0.2%)	
2	129 (28.7%)	120 (26.8%)	
3	15 (3.3%)	7 (1.6%)	
4	260 (57.9%)	273 (60.9%)	
5	22 (4.9%)	24 (5.4%)	
**Randomized assignment−*n* (%)**			
Intensive blood pressure lowering	222 (49.4%)	230 (51.3%)	0.57
Standard blood pressure lowering	227 (50.6%)	218 (48.7%)	
**Received surgical treatment−*n* (%)**			
Intraventricular catheter placed	27 (6.0%)	29 (6.5%)	0.68
Surgical hematoma evacuation	15 (3.3%)	19 (4.2%)	0.55
**Long-term disability assessment by mRS−*n* (%)^d^**			
0–1	124 (27.6%)	105 (23.4%)	0.39
2–3	148 (33.0%)	167 (37.3%)	
4–5	137 (30.5%)	131 (29.2%)	
6	27 (6.0%)	33 (7.4%)	
Unknown	13 (2.9%)	12 (2.7%)	

^a^Hematoma expansion was defined as an ICH volume increase > 33% or > 6 ml from baseline to 24-h follow-up non-contrast CT.

^b^Values are from original images before pre-processing.

^c^Binary variables were obtained by majority vote of the three reads. Diagnostic criteria were adopted from Morotti et al. (2019).

^d^mRS score at 90 days after randomization; if unavailable, mRS assessments from (1) > 90 days and (2) > 30 and < 90 days after randomization were utilized as first and second alternatives, respectively. CT, computed tomography; GCS, Glasgow Coma Scale; ICH, intracerebral hemorrhage; mRS, modified Rankin Scale; NIHSS, National Institutes of Health Stroke Scale; SD, standard deviation; TIA, transient ischemic attack.

### Signatures of HE

The radiomics signature consisted of two first-order, one shape, and three texture features, and predicted HE with an AUC (95% CI) of 0.64 (0.59–0.70) and 0.61 (0.56–0.67) in the discovery and independent validation cohort, respectively. The visual signature incorporated six visual markers of HE, with the “swirl sign,” “black hole sign,” and “irregular shape” weighted the strongest. The visual signature attained an AUC (95% CI) of 0.59 (0.53–0.65) and 0.57 (0.51–0.63) in the discovery and independent validation cohort, respectively. The clinical signature, consisting of only the baseline NIHSS score, reached an AUC (95% CI) of 0.61 (0.55–0.66) and 0.57 (0.51–0.63) in the discovery and validation cohort, respectively. The BAT score alone achieved an AUC (95% CI) of 0.54 (0.49–0.60) and 0.54 (0.49–0.59) in the discovery and validation cohort, respectively.

In generating the radiomics + visual and the radiomics + BAT signatures, the LASSO-LR model selected neither visual markers nor the BAT score. Therefore, the signatures’ composition and performance defaulted to the radiomics signature, indicating visual markers of HE and the BAT score provide no added predictive value. The radiomics + clinical signature’s composition and performance closely resembled that of the radiomics signature, with only one clinical variable (NIHSS score) incorporated. No visual markers were included in the radiomics + visual + clinical signature, and the signature’s composition and performance defaulted to the radiomics + clinical signature, again indicating visual markers provide no added predictive value.

Among combined signatures generated by supplying select radiomic features to LASSO-LR models as candidate variables, only the select radiomics + clinical signature’s AUC in the validation cohort differed from corresponding baseline signatures’ AUC generated by supplying all radiomic features. The select radiomics + clinical signature was the strongest predictor of HE overall, with an AUC (95% CI) of 0.67 (0.61–0.72) and 0.64 (0.59–0.70) in the discovery and validation cohort, respectively. It incorporated the same *n* = 6 radiomic features as the radiomic signature and all clinical variables.

Table 2 depicts signatures’ performance in both cohorts; the signatures’ composition with corresponding regression coefficients is reported in Supplementary Table 3. Supplementary Table 4 provides definitions of radiomic features included in signatures.

**TABLE 2:** Performance of signatures in predicting hematoma expansion.

	Discovery cohort^a^			Independent validation cohort	
	**Mean CV AUC (SE)**	**AUC** **(95% CI)^b^**	**Precision/** **Recall/NPV/F1^d^**	**AUC** **(95% CI)^b^**	**Precision/** **Recall/NPV/F1^d^**
Radiomics signature	0.61 (0.03)	0.64 (0.59–0.70)	0.31/0.81/0.84/0.45	0.61 (0.56–0.67)	0.32/0.75/0.80/0.46
Visual signature	0.55 (0.02)	0.59 (0.53–0.65)	0.29/0.81/0.81/0.43	0.57 (0.51–0.63)	0.31/0.76/0.78/0.44
Clinical signature	0.61 (0.02)	0.61 (0.55–0.66)	0.30/0.82/0.83/0.44	0.57 (0.51–0.63)	0.29/0.77/0.75/0.42
BAT score	n/a	0.54 (0.49–0.60)	0.27/0.98/0.91/0.43	0.54 (0.49–0.59)	0.29/0.98/0.88/0.45
Radiomics + visual signature	0.61 (0.03)	0.64 (0.59–0.70)	0.31/0.81/0.84/0.45	0.61 (0.56–0.67)	0.33/0.75/0.80/0.46
Radiomics + clinical signature	0.60 (0.03)	0.65 (0.59–0.70)	0.32/0.81/0.84/0.45	0.62 (0.57–0.68)	0.33/0.81/0.83/0.47
Radiomics + BAT signature	0.60 (0.03)	0.64 (0.59–0.70)	0.31/0.81/0.84/0.45	0.61 (0.56–0.67)	0.33/0.75/0.80/0.46
Select radiomics^c^ + visual signature	0.63 (0.03)	0.65 (0.59–0.71)	0.31/0.81/0.84/0.45	0.61 (0.56–0.67)	0.34/0.79/0.83/0.47
Select radiomics^c^ + clinical signature	0.63 (0.03)	0.67 (0.61–0.72)	0.33/0.81/0.86/0.47	0.64 (0.59–0.70)	0.34/0.81/0.84/0.48
Radiomics + visual + clinical signature	0.60 (0.03)	0.65 (0.59–0.70)	0.32/0.81/0.84/0.45	0.62 (0.57–0.68)	0.33/0.81/0.83/0.47
Select radiomics^c^ + visual + clinical signature	0.62 (0.03)	0.65 (0.59–0.71)	0.32/0.81/0.85/0.46	0.62 (0.57–0.68)	0.34/0.81/0.83/0.47

^a^The left column shows average test fold AUCs and corresponding SEs across k-fold stratified CV (*k* = 10, strata: HE-positive and -negative subpopulations) obtained by the “cv.glmnet” R function using optimized lambda parameters; the middle and right column depict final signatures’ performance in the total discovery cohort.

^b^DeLong’s method was applied to calculate 95% CIs (DeLong et al., 1988).

^c^Only radiomics features included in the radiomics signature were supplied to LASSO-LR models.

^d^The threshold against which continuous signature scores were dichotomized was selected to attain a recall of 0.8 or greater in the discovery cohort. The F1-score is the harmonic mean of the precision and recall. AUC, area under the receiver operating characteristic curve; CI, confidence interval; CV, cross validation; HE, hematoma expansion; LASSO-LR, least absolute shrinkage and selection operator-regularized logistic regression; NPV, negative predictive value, SE, standard error.

A pipeline iteration adding ComBat harmonization of radiomic features to mitigate batch effects of CT slice thickness yielded slightly numerically improved results in the discovery cohort, but numerically inferior AUCs in independent validation (Supplementary Table 5).

### Comparison of signatures’ performance in predicting HE

The select radiomics + clinical signature attained the highest AUC scores and outperformed the visual signature, clinical signature and BAT score in the discovery cohort (*p* = 0.03, *p* = 0.02, *p* < 0.001, respectively, DeLong’s test, Table 3) and the independent validation cohort (*p* = 0.01, *p* = 0.01, *p* < 0.001, respectively). In addition, its AUC was significantly higher than the radiomics signature’s in the validation cohort (*p* = 0.04), with *p* = 0.11 in the discovery cohort. Moreover, all signatures incorporating radiomic features achieved significantly higher AUCs than the BAT score in both cohorts (all *p* < 0.05), while the visual and clinical signatures did not (all *p* > 0.05).

**TABLE 3:** Comparison of signatures’ performance in predicting hematoma expansion.

DeLong’s test^a^	Radiomics	Visual	Clinical	BAT score	Radiomics + visual	Radiomics + clinical	Radiomics + BAT	Select radiomics^b^ + visual	Select radiomics^b^ + clinical	Radiomics + visual + clinical
**Discovery cohort**										
**Visual**	*p* = 0.16									
**Clinical**	*p* = 0.23	*p* = 0.73								
**BAT score**	***p* = *0.005***	*p* = 0.06	*p* = 0.07							
**Radiomics + visual**	*p* = 1.00	*p* = 0.16	*p* = 0.23	***p* = *0.005***						
**Radiomics** + **clinical**	*p* = 0.75	*p* = 0.13	*p* = 0.09	***p* = *0.003***	*p* = 0.75					
**Radiomics** + **BAT**	*p* = 1.00	*p* = 0.16	*p* = 0.23	***p* = *0.005***	*p* = 1.00	*p* = 0.75				
**Select radiomics**^**b**^ + **visual**	*p* = 0.25	*p* = 0.11	*p* = 0.16	***p* = *0.002***	*p* = 0.25	*p* = 0.80	*p* = 0.25			
**Select radiomics**^**b**^ + **clinical**	*p* = 0.11	***p* = *0.03***	***p* = *0.02***	***p* = *0.0004***	*p* = 0.11	*p* = 0.10	*p* = 0.11	*p* = 0.17		
**Radiomics** + **visual** + **clinical**	*p* = 0.75	*p* = 0.13	*p* = 0.09	***p* = *0.003***	*p* = 0.75	*p* = 1.00	*p* = 0.75	*p* = 0.80	*p* = 0.10	
**Select radiomics**^**b**^ + **visual** + **clinical**	*p* = 0.53	*p* = 0.12	*p* = 0.08	***p* = *0.003***	*p* = 0.53	*p* = 0.36	*p* = 0.53	*p* = 1.00	*p* = 0.14	*p* = 0.36
**Independent validation cohort**										
**Visual**	*p* = 0.18									
**Clinical**	*p* = 0.16	*p* = 0.92								
**BAT score**	***p* = *0.02***	*p* = 0.21	*p* = 0.36							
**Radiomics** + **visual**	*p* = 1.00	*p* = 0.18	*p* = 0.16	***p* = *0.02***						
**Radiomics** + **clinical**	*p* = 0.30	*p* = 0.07	***p* = *0.02***	***p* = *0.008***	*p* = 0.30					
**Radiomics** + **BAT**	*p* = 1.00	*p* = 0.18	*p* = 0.16	***p* = *0.02***	*p* = 1.00	*p* = 0.30				
**Select radiomics**^**b**^ + **visual**	*p* = 0.47	*p* = 0.15	*p* = 0.14	***p* = *0.02***	*p* = 0.47	*p* = 0.46	*p* = 0.47			
**Select radiomics**^**b**^ + **clinical**	***p* = *0.04***	***p* = *0.01***	***p* = *0.01***	***p* = *0.0006***	***p* = *0.04***	*p* = 0.18	***p* = *0.04***	*p* = 0.05		
**Radiomics** + **visual** + **clinical**	*p* = 0.30	*p* = 0.07	***p* = *0.02***	***p* = *0.008***	*p* = 0.30	*p* = 1.00	*p* = 0.30	*p* = 0.46	*p* = 0.18	
**Select radiomics**^**b**^ + **visual** + **clinical**	*p* = 0.29	*p* = 0.08	***p* = *0.03***	***p* = *0.009***	*p* = 0.29	*p* = 0.61	*p* = 0.29	*p* = 0.46	*p* = 0.14	*p* = 0.61

^a^DeLong’s test was applied to compare AUC scores (DeLong et al., 1988).

^b^Only radiomic features included in the radiomics signature were supplied to LASSO-LR models. AUC, area under the receiver operating characteristic curve; LASSO-LR, least absolute shrinkage and selection operator-regularized logistic regression.

Bold and italic values indicate a significant *p*-value.

### Association of signatures with long-term functional outcome

All signatures were significantly correlated with the mRS score in both the discovery and independent validation cohort, with Spearman’s rho ranging from *r* = 0.22 to *r* = 0.58 (all *p* < 0.001, Table 4).

**TABLE 4:** Association of signatures with long-term functional outcome.

Correlation with 3-month mRS score	Spearman’s rho (95% CI)	*p*-value
**Discovery cohort**		
Radiomics signature	0.30 (0.21–0.38)	*p* < 0.001
Visual signature	0.25 (0.16–0.33)	*p* < 0.001
Clinical signature	0.58 (0.51–0.64)	*p* < 0.001
BAT score	0.22 (0.13–0.31)	*p* < 0.001
Radiomics + visual signature	0.30 (0.21–0.38)	*p* < 0.001
Radiomics + clinical signature	0.43 (0.36–0.51)	*p* < 0.001
Radiomics + BAT signature	0.30 (0.21–0.38)	*p* < 0.001
Select radiomics^a^ + visual signature	0.30 (0.21–0.39)	*p* < 0.001
Select radiomics^a^ + clinical signature	0.37 (0.28–0.45)	*p* < 0.001
Radiomics + visual + clinical signature	0.43 (0.36–0.51)	*p* < 0.001
Select radiomics^a^ + visual + clinical signature	0.42 (0.34–0.49)	*p* < 0.001
**Independent validation cohort**		
Radiomics signature	0.33 (0.25–0.42)	*p* < 0.001
Visual signature	0.40 (0.31–0.47)	*p* < 0.001
Clinical signature	0.56 (0.49–0.62)	*p* < 0.001
BAT score	0.26 (0.17–0.34)	*p* < 0.001
Radiomics + visual signature	0.33 (0.25–0.42)	*p* < 0.001
Radiomics + clinical signature	0.46 (0.38–0.53)	*p* < 0.001
Radiomics + BAT signature	0.33 (0.25–0.42)	*p* < 0.001
Select radiomics^a^ + visual signature	0.32 (0.24–0.41)	*p* < 0.001
Select radiomics^a^ + clinical signature	0.35 (0.26–0.43)	*p* < 0.001
Radiomics + visual + clinical signature	0.46 (0.38–0.53)	*p* < 0.001
Select radiomics^a^ + visual + clinical signature	0.44 (0.36–0.51)	*p* < 0.001

^a^Only radiomic features included in the radiomics signature were supplied to LASSO-LR models. CI, confidence interval; LASSO-LR, least absolute shrinkage and selection operator-regularized logistic regression; mRS, modified Rankin Scale.

## Discussion

Using a large, multicentric cohort of patients with acute, spontaneous, supratentorial ICH, we devised and validated radiomic signatures for prediction of ICH expansion using features from baseline non-contrast head CT scans. Given that participants were prospectively enrolled in the ATACH-2 trial under controlled conditions, our dataset offers accurate clinical information as well as precisely timed baseline and 24-h follow-up scans enabling rigorous design and validation of HE prediction models. In an independent validation cohort, we demonstrated that a signature combining select radiomic with clinical features of ICH was significantly superior to signatures of visual markers of HE, clinical variables associated with HE, the BAT score and a radiomics-only signature (all *p* < 0.05). In addition, one should consider the reliability of an automatically extracted radiomic signature versus the complexity of visual assessment of six different HE markers in acute ICH settings. Future studies may combine deep learning hematoma segmentation (Dhar et al., 2020) with radiomics to enable fully automated HE prediction and further reduce reader-dependency. Notably, the fact that neither the BAT score nor visual markers were retained in combined signatures (Supplementary Table 3) suggests that they provide no added predictive value over radiomic features. Moreover, the visual signature yielded numerically but not significantly higher AUCs than the BAT score in both cohorts, suggesting a more comprehensive visual scoring system might yield improved prediction results at the expense of longer and more complex visual image interpretation (Tables 2, 3). Finally, we confirmed the clinical relevance of HE signatures for prognostication of functional outcome by showing consistent associations with 3-month mRS score in the discovery and validation cohorts (Table 4).

Hematoma growth is strongly associated with poor functional outcome and mortality in ICH patients, and therefore, attenuation of ICH expansion is considered a potential treatment strategy (Davis et al., 2006). Unfortunately, thus far, neither intensive blood pressure reduction (Anderson et al., 2013; Qureshi et al., 2016), nor administration of hemostatic drugs such as recombinant factor VII (Mayer et al., 2008) or tranexamic acid (Sprigg et al., 2018; Meretoja et al., 2020), which theoretically target HE, could reduce death or disability in randomized clinical trials. In addition, trials evaluating selective hemostatic therapy of CT-A spot sign-positive patients failed to demonstrate significant treatment benefits (Gladstone et al., 2019; Meretoja et al., 2020). Hence, the search for an effective ICH therapy and (imaging) biomarkers for treatment triage remains ongoing. In this context, an objective and reproducible marker of impending HE based on admission non-contrast head CT−which is readily available and widely used as first-line imaging in emergency departments−may allow future clinical trials to selectively enroll patients who likely benefit from anti-expansion therapy and may ultimately improve ICH outcomes.

In our study, we allocated and strictly separated discovery and independent validation cohorts to accurately quantify radiomic signatures’ performance in predicting HE. Signatures attained very similar AUC scores in both cohorts as well as in cross validation, which is indicative of reliable generalizability. In terms of absolute performance compared to previous studies, our radiomic signature results are similar to those of e.g., Pszczolkowski et al. (2021), who also conducted *post hoc* analyses of randomized controlled trial data, with an identical HE definition, and similar methodology. On the other hand, Xie et al. (2020), who likewise applied LASSO-LR to devise radiomic signatures, reported AUCs of up to 0.93 in independent validation. The difference in AUC score may be in part attributed to the use of an identical scanner and imaging protocol for all patients by Xie et al. (2020). In addition, the average baseline ICH volume in the study by Xie et al. (2020) was ∼32 ml in patients with HE and ∼12.5 to 14 ml in patients without HE (*p* < 0.001), suggesting that volume by itself was highly predictive of HE. In our data, however, the baseline volumes differed conspicuously less, with a mean (SD) volume of 13.7 ml (12.7) and 12.2 ml (11.7) among patients with and without HE, respectively (*p* = 0.13, Wilcoxon rank sum test). As a result, our signatures could not exploit the volume differences. In general, critical appraisal of study populations, methodology, and validation is warranted when comparing radiomics research, where overfitting and information leakage from discovery to validation datasets are frequently encountered challenges.

Multiple visual makers on non-contrast CT were proposed as predictors of ICH expansion (Boulouis et al., 2017; Morotti et al., 2018, 2019). However, overlapping definitions and subjective interpretations may limit their reproducibility. Radiomics, on the other hand, offers reproducible, quantitative, and objective metrics of ICH size, shape, intensity, and heterogeneity characteristics. In this study, we demonstrated that visual markers−alone or in combination−provide no added predictive value to radiomic signatures in prediction of ICH expansion (Supplementary Table 3 and Table 2). In addition, our radiomics-based signatures significantly outperformed the visual signature and BAT score when combined with select clinical predictors. Overall, the objectivity and rapid applicability of radiomic signatures could make them suitable triage tools for multicentric randomized controlled trials, where observer-independent and expeditious enrollment is crucial.

We utilized a large, multicentric, multi-national, prospectively acquired and homogeneous patient dataset with accurately timed baseline and follow-up CT imaging and comprehensive clinical data gathered by a randomized clinical trial under strict oversight, as opposed to previous studies which often relied on retrospective single-center data collection. In addition, we employed state-of-the-art radiomic analysis and strictly separated discovery and validation cohorts to prevent information leakage and performance inflation. The ATACH-2 enrollment criteria, however, inherently limit our findings to patients with acute, spontaneous, supratentorial ICH, hypertension, and a baseline hematoma volume < 60 cm^3^ (Qureshi et al., 2016). Further studies in more inclusive cohorts are needed to validate our radiomic signatures. Moreover, future studies may compare the predictive value of our signatures with the CT-A spot sign. In addition, although radiomics signatures had significant association with 3-month clinical outcomes, improvement of radiomic HE biomarkers’ absolute predictive performance is crucial before routine clinical application or clinical trials may be considered. It is worth noting that non-contrast head CTs are among the most harmonized medical images: the uniform use of soft tissue kernels, absence of intravenous contrast administration, and calibration of Hounsfield units to exact physical density obviate the need for gray scale normalization. To mitigate the effects of slice thickness and voxel dimension variability, we applied B-spline interpolation to resample images to an isotropic 1 mm × 1 mm × 1 mm voxel spacing, as detailed in the Supplementary methods. To further mitigate the effects of CT slice thickness differences on radiomic feature values, we applied ComBat harmonization. However, while achieving a slight numeric improvement in prediction accuracy within the discovery cohort, signatures compiled from harmonized radiomic features yielded numerically inferior AUCs in the validation cohort, which may be indicative of overfitting (compare Table 2 and Supplementary Table 5). There is still ample potential for refinements, which may include further harmonizing CT imaging protocols across centers, usage of higher resolution scans and reconstructions, automated segmentation algorithms and incorporation of radiomic features from additional ICH manifestations such as the perilesional edema or intraventricular hemorrhage. Nevertheless, we believe our study, confirming the results of some prior reports, underlines the value of radiomics in HE prediction.

## Conclusion

Using a large multicentric dataset, we generated and independently validated a radiomic signature of HE based on admission non-contrast head CTs of patients with supratentorial ICH. We demonstrated that a signature combining radiomic features and clinical predictors significantly outperforms a signature of visual CT markers of HE as well as the BAT score, and that adding visual markers to radiomic features offers no improvement in predictive performance. All HE signatures were significantly associated with 3-month functional outcome, underlining their prognostic relevance. Limited to ICH patients with similar characteristics, the proposed markers may enable selective anti-expansion treatment of patients at higher risk of HE in future clinical trials.

## Data availability statement

The data analyzed in this study is subject to the following licenses/restrictions: data are available on reasonable request, and on approval from the respective register holders. Requests to access these datasets should be directed to the ATACH-2 investigators (ClinicalTrials.gov identifier: NCT01176565).

## Ethics statement

Ethical compliance was ensured by the ATACH-2 investigators (ClinicalTrials.gov identifier: NCT01176565). Our group performed *post hoc* analyses of anonymized data. The patients/participants provided their written informed consent to participate in this study.

## Author contributions

SH: conceptualization, data curation, formal analysis, investigation, methodology, project administration, software, validation, visualization, writing—original draft, writing—review and editing. AQ, AJ, HT, and EB: data curation, investigation writing—review and editing. TZ: formal analysis, investigation, writing—review and editing. DW, MG, AdM, AjM, LS, GF, and KS: investigation, writing—review and editing. SP: conceptualization, data curation, formal analysis, funding acquisition, investigation, methodology, project administration, resources, software, supervision, validation, writing—original draft, writing—review and editing. All authors contributed to the article and approved the submitted version.
