# Supplementary material for: Radiomic markers of intracerebral hemorrhage expansion on non-contrast CT: independent validation and comparison with visual markers
Source: Front Neurosci. 2023 Aug 16;17:1225342. doi: 10.3389/fnins.2023.1225342 (PMC10467422; doi:10.3389/fnins.2023.1225342)
Supplement: Supplementary file 1 [file Data_Sheet_1.pdf]

SUPPLEMENTAL MATERIAL

for the original article

Radiomic markers of intracerebral hemorrhage expansion on non-contrast CT: independent validation and comparison with visual markers

Contents

1. Supplemental methods 3

Image pre-processing and radiomics feature extraction pipeline 3

Generation of hematoma expansion signatures using LASSO-LR.. 3

Radiomic feature harmonization 3

Radiomics feature selection (intra- and inter-rater stability, inter-feature collinearity) 4

2. Supplemental tables.. 5

Supplemental table 1 List of extracted radiomic features. 5

Supplemental table 2 Diagnostic criteria for visual non-contrast CT markers of hematoma expansion 8

Supplemental table 3 Signatures of hematoma expansion. 9

Supplemental table 4 Definitions of radiomic features included in signatures. 12

Supplemental table 5 Performance of signatures compiled from harmonized radiomic features. 13

3. Supplemental references 14

1. Supplemental methods

Image pre-processing and radiomics feature extraction pipeline

The below section was originally included in the supplement for [1] and is reproduced here to support comprehensibility of the manuscript.

In order to minimize the effects of data heterogeneity and differences in voxel dimensions on the radiomics extraction [2, 3], CT images and masks were resampled to an isotropic 1x1x1 mm voxel spacing using "B-spline" interpolation [4-6]. Interpolation to isotropic voxels ensures rotational invariance of texture features [7]. Then, we removed voxels outside the 1-200 Hounsfield unit (HU) range from ICH masks ("re-segmentation") to restrict analysis to a HU range encompassing brain tissue and ICH densities, but excluding dense parenchymal calcifications or osseous structures. Finally, original CT images were filtered to refine analysis of certain hematoma characteristics [4]: By applying high- and low-pass filters in each spatial direction, we generated eight decompositions per original image ("coif-1" wavelet transform) [8, 9]. An "edge-enhancement" Laplacian of Gaussian (LoG) filter with "sigma" settings of 2 mm, 4 mm and 6 mm yielded three additional derivative images per original [9, 10]. "First-order" and "texture-matrix" radiomics feature extraction requires an image grey scale discretization step in pre-processing ("binning") [7], which was implemented using a fixed bin width method with the width parameter set to 2 HU [9]. A complete list of radiomics features utilized in this study is provided in supplemental table 1. "Shape" features (n=14 features) were extracted from hematoma representations in the original images; "first-order" (n=18) and "texture-matrix" features (n=75) were extracted from original images and eleven derivative images per original (eight "coif-1" wavelet- and three LoG filtering-derivates). This approach yielded a total of n=1130 radiomics features per subject. We customized a Pyradiomics version 2.1.2 pipeline to facilitate pre-processing, derivative image generation including wavelet transforms and LoG filtering, and feature extraction [9, 11].

Generation of hematoma expansion signatures using LASSO-LR

We utilized the "cv.glmnet" function (R "glmnet" version 2.0-18 package) to fit and analyze least absolute shrinkage and selection operator-regularized logistic regression (LASSO-LR) models (function arguments: type.measure = "auc", family = "binomial", alpha = 1) [12]. The function automatically optimized the LASSO lambda parameter prior to final model fitting by testing a sequence of lambda values in stratified k-fold cross validation performed solely on the discovery cohort (k = 10, strata: HE-positive and -negative subpopulations); the lambda value maximizing the area under the receiver operating characteristic curve (AUC) averaged across validation folds was selected. After fitting a final model on the entire discovery cohort, the regression coefficients of candidate variables were queried, candidate variables with regression coefficients equal to zero were excluded, and the linear combination of the remaining variables weighted by their respective coefficients was retained as a "signature".

Radiomic feature harmonization

We harmonized each radiomic feature separately applying parametric ComBat harmonization to mitigate batch effects of CT slice thickness on radiomic features ("neuroCombat" R function from "neuroCombat" version 1.0.13 package; function arguments: eb = FALSE, parametric = TRUE; the remaining parameters were set to the default package recommendations) [13, 14]. Using the slice thickness from original head CT images (i.e., prior to voxel dimension resampling) as the batching

variable, we created three batches, similarly to Pszczolkowski et al. [15]: slice thickness ≤ 3mm, 3mm < slice thickness ≤ 6mm, and slice thickness > 6mm. To preclude data leakage from the discovery to the validation cohort, we estimated ComBat parameters from the discovery cohort only, and subsequently harmonized the validation cohort applying the same parameters ("neuroCombatFromTraining" R function from "neuroCombat" version 1.0.13 package).

Radiomics feature selection (intra- and inter-rater stability, inter-feature collinearity)

The below sections were originally included in the supplement for [1] and are reproduced here to support comprehensibility of the manuscript.

The robustness of individual radiomics features to intra- and inter-rater segmentation inconsistencies was investigated in a set of n=100 patients ("multiple delineation cohort") who were randomly sampled from the discovery cohort. The hematomas in these patients were segmented a second and third time by the original reviewer and an additional reviewer, respectively [4]. Subsequently, we extracted radiomics features from all three segmentation sets and calculated intra-/interclass correlation coefficient (ICC) statistics to assess the intra-/inter-rater agreement of each radiomics feature [4]: a "two-way mixed effects, absolute agreement, single rater/measurement ICC" was utilized to quantify intra-rater agreement; and a "two-way random effects, absolute agreement, single rater/measurement ICC" was applied to assess the inter-rater agreement [16, 17]. Features with an ICC 95% confidence interval lower bound ≥ 0.8 in both intra- and inter-rater assessment were considered robust. In total, 1002/1130 (88.7 %) radiomics features met this criterion and were retained for further analysis. The R "psych" package "ICC" function was applied to compute ICC statistics [18].

To reduce collinearity, we generated a radiomics feature correlation matrix using the discovery cohort and Spearman's correlation coefficient (rho) as the correlation metric (R "stats" package "cor" function) [19]. From any correlated feature pair with |rho| > 0.95, the feature with higher mean absolute correlation across the discovery cohort was excluded (R "caret" package "findCorrelation" function) [20]. A total of 429/1002 (42.8 %) features were retained in the collinearity-reduced feature set.

2. Supplemental tables

Supplemental table 1 List of extracted radiomic features.

Feature Family		Feature name
**First-order**	1	10th percentile
	2	90th percentile
	3	Energy
	4	Entropy
	5	Interquartile Range
	6	Kurtosis
	7	Maximum
	8	Mean
	9	Mean Absolute Deviation
	10	Median
	11	Minimum
	12	Range
	13	**Robust Mean Absolute Deviation** [a]
	14	Root Mean Squared
	15	**Skewness** [a]
	16	Total Energy
	17	Uniformity
	18	Variance
**Shape**	1	Elongation
	2	Flatness
	3	Least Axis Length
	4	Major Axis Length
	5	Maximum 2D Diameter Column
	6	Maximum 2D Diameter Row
	7	**Maximum 2D Diameter Slice** [a]
	8	Maximum 3D Diameter
	9	Mesh Volume
	10	Minor Axis Length
	11	Sphericity
	12	Surface Area
	13	Surface Area to Volume Ratio
	14	Voxel Volume
**Texture - Gray Level Cooccurrence Matrix Features (glcm)**	1	Autocorrelation
	2	Cluster Prominence
	3	**Cluster Shade** [a]
	4	Cluster Tendency
	5	Contrast
	6	Correlation
	7	Difference Average
	8	Difference Entropy

	9	Difference Variance
	10	Informational Measure of Correlation 1
	11	Informational Measure of Correlation 2
	12	Inverse Difference
	13	Inverse Difference Moment
	14	Inverse Difference Moment Normalized
	15	Inverse Difference Normalized
	16	Inverse Variance
	17	Joint Average
	18	Joint Energy
	19	Joint Entropy
	20	Maximal Correlation Coefficient
	21	Maximum Probability
	22	Sum Average
	23	Sum Entropy
	24	Sum of Squares
**Texture - Gray Level Size Zone Matrix Features (glszm)**	1	Gray Level Non-Uniformity
	2	Gray Level Non-Uniformity Normalized
	3	Gray Level Variance
	4	High Gray Level Zone Emphasis
	5	Large Area Emphasis
	6	Large Area High Gray Level Emphasis
	7	**Large Area Low Gray Level Emphasis** [a]
	8	Low Gray Level Zone Emphasis
	9	Size Zone Non-Uniformity
	10	Size Zone Non-Uniformity Normalized
	11	**Small Area Emphasis** [a]
	12	Small Area High Gray Level Emphasis
	13	Small Area Low Gray Level Emphasis
	14	Zone Entropy
	15	Zone Percentage
	16	Zone Variance
**Texture - Gray Level Run Length Matrix Features (glrlm)**	1	Gray Level Non-Uniformity
	2	Gray Level Non-Uniformity Normalized
	3	Gray Level Variance
	4	High Gray Level Run Emphasis
	5	Long Run Emphasis
	6	Long Run High Gray Level Emphasis
	7	Long Run Low Gray Level Emphasis
	8	Low Gray Level Run Emphasis
	9	Run Entropy
	10	Run Length Non-Uniformity
	11	Run Length Non-Uniformity Normalized
	12	Run Percentage

	13	Run Variance
	14	Short Run Emphasis
	15	Short Run High Gray Level Emphasis
	16	Short Run Low Gray Level Emphasis
**Texture - Neighboring Gray Tone Difference Matrix Features (ngtdm)**	1	Busyness
	2	Coarseness
	3	Complexity
	4	Contrast
	5	Strength
**Texture - Gray Level Dependence Matrix Features (gldm)**	1	Dependence Entropy
	2	Dependence Non-Uniformity
	3	Dependence Non-Uniformity Normalized
	4	**Dependence Variance** [a]
	5	Gray Level Non-Uniformity
	6	Gray Level Variance
	7	High Gray Level Emphasis
	8	Large Dependence Emphasis
	9	Large Dependence High Gray Level Emphasis
	10	Large Dependence Low Gray Level Emphasis
	11	Low Gray Level Emphasis
	12	Small Dependence Emphasis
	13	Small Dependence High Gray Level Emphasis
	14	Small Dependence Low Gray Level Emphasis

[a] Indicates the feature was included in a signature (see supplemental table 3).

List of Pyradiomics [11] features utilized in this study. Exact feature definitions are provided in ref. [9].

Supplemental table 2 Diagnostic criteria for visual non-contrast CT markers of hematoma expansion

Visual marker	Diagnostic criteria [a]
**Black hole sign**	“Hypoattenuating area with a density difference >28HU compared with the surrounding hematoma. No connection with surface outside the hematoma.” [21]
**Blend sign**	“Relatively hypoattenuating area next to a hyperattenuating area of the hematoma, with a well-defined margin and a density difference >18HU between the 2 areas.” [21]
**Fluid level**	“Presence of 1 distinct hypoattenuating area (hypodense to the brain) above and 1 hyperattenuating area (hyperdense to the brain) below a discrete straight line of separation, irrespective of its density appearance.” [21]
**Hypodensity**	“Any hypodense region strictly encapsulated within the hemorrhage with any shape, size, and density. Does not require density measurement.” [21]
**Irregular shape**	“2 or more focal hematoma margin irregularities, joined or separate from the hematoma edge, evaluated on the axial NCCT slice showing the largest ICH area (Barras shape scale = III, IV, or V).” [21]
**Island sign**	“At least 3 scattered small hematomas all separate from the main ICH or at least 4 small hematomas some or all of which may connect with the ICH.” [21]
**Satellite sign**	“A small hematoma (diameter < 10mm) separate from the main hemorrhage in at least 1 slice and distinct from main hematoma by 1–20mm separation.” [21]
**Swirl sign**	“Rounded, streaklike, or irregular region of hypo- or isoattenuation compared with the brain parenchyma. Does not have to be encapsulated in the ICH.” [21]

[a] Diagnostic criteria for visual non-contrast head CT markers of hematoma expansion were reproduced from Morotti et al. [21].

Supplemental table 3 Signatures of hematoma expansion.

Supplemental table 3.1 Radiomics signature.

Feature identifier [a]				Coefficient [b]
Pre-processing		Family	Feature name	
wavelet	LLL	first-order	Robust mean absolute deviation	-0.152
original	n/a	shape	Maximum 2D diameter slice	0.133
original	n/a	glszm	Large area low gray level emphasis	0.015
LoG	6 mm	first-order	Skewness	0.013
LoG	6 mm	gldm	Dependence variance	0.007
wavelet	LLL	glcm	Cluster shade	0.001

Supplemental table 3.2 Visual signature.

Visual marker	Coefficient [b]
Swirl sign	0.525
Black hole sign	0.300
Irregular shape	0.216
Island sign	0.172
Time from symptom onset to baseline CT	-0.157
Hypodensity	0.134
Satellite sign	0.106

Supplemental table 3.3 Clinical signature.

Clinical variable	Coefficient [b]
NIHSS score at baseline	0.059

Supplemental table 3.4 Radiomics + visual signature.

Feature identifier [a] or visual marker				Coefficient [b]
Pre-processing		Family	Feature name	
wavelet	LLL	first-order	Robust mean absolute deviation	-0.152
original	n/a	shape	Maximum 2D diameter slice	0.133
original	n/a	glszm	Large area low gray level emphasis	0.015
LoG	6 mm	first-order	Skewness	0.013
LoG	6 mm	gldm	Dependence variance	0.007
wavelet	LLL	glcm	Cluster shade	0.001

Supplemental table 3.5 Radiomics + clinical signature.

Feature identifier [a] or clinical variable				Coefficient [b]
Pre-processing		Family	Feature name	
wavelet	LLL	first-order	Robust mean absolute deviation	-0.139
NIHSS score at baseline				0.093
original	n/a	shape	Maximum 2D diameter slice	0.082

original	n/a	glszm	Large area low gray level emphasis	0.018
LoG	6 mm	gldm	Dependence variance	0.012
LoG	6 mm	glszm	Small area emphasis	0.010
LoG	6 mm	first-order	Skewness	0.003
wavelet	LLL	glcm	Cluster shade	0.002

Supplemental table 3.6 Radiomics + BAT signature.

Feature identifier [a]				Coefficient [b]
Pre-processing		Family	Feature name	
wavelet	LLL	first-order	Robust mean absolute deviation	-0.152
original	n/a	shape	Maximum 2D diameter slice	0.133
original	n/a	glszm	Large area low gray level emphasis	0.015
LoG	6 mm	first-order	Skewness	0.013
LoG	6 mm	gldm	Dependence variance	0.007
wavelet	LLL	glcm	Cluster shade	0.001

Supplemental table 3.7 Select radiomics [c] + visual signature.

Feature identifier [a] or visual marker				Coefficient [b]
Pre-processing		Family	Feature name	
wavelet	LLL	first-order	Robust mean absolute deviation	-0.298
original	n/a	shape	Maximum 2D diameter slice	0.228
wavelet	LLL	glcm	Cluster shade	0.124
LoG	6 mm	first-order	Skewness	0.071
original	n/a	glszm	Large area low gray level emphasis	0.064
Time from symptom onset to baseline CT				-0.032

Supplemental table 3.8 Select radiomics [c] + clinical signature.

Feature identifier [a] or clinical variable				Coefficient [b]
Pre-processing		Family	Feature name	
wavelet	LLL	glcm	Cluster shade	1.512
Sex				0.356
wavelet	LLL	first-order	Robust mean absolute deviation	-0.319
NIHSS score at baseline				0.221
original	n/a	shape	Maximum 2D diameter slice	0.185
LoG	6 mm	first-order	Skewness	0.100
GCS score at baseline				0.096
original	n/a	glszm	Large area low gray level emphasis	0.091
Blood glucose at baseline				0.082
Platelet count at baseline				0.063
Time from symptom onset to baseline CT				-0.062
LoG	6 mm	gldm	Dependence variance	-0.012

Supplemental table 3.9 Radiomics + visual + clinical signature.

Feature identifier [a] or visual marker or clinical variable				Coefficient [b]
Pre-processing		Family	Feature name	
wavelet	LLL	first-order	Robust mean absolute deviation	-0.139
NIHSS score at baseline				0.093
original	n/a	shape	Maximum 2D diameter slice	0.082
original	n/a	glszm	Large area low gray level emphasis	0.018
LoG	6 mm	gldm	Dependence variance	0.012
LoG	6 mm	glszm	Small area emphasis	0.010
LoG	6 mm	first-order	Skewness	0.003
wavelet	LLL	glcm	Cluster shade	0.002

Supplemental table 3.10 Select radiomics [c] + visual + clinical signature.

Feature identifier [a] or visual marker or clinical variable				Coefficient [b]
Pre-processing		Family	Feature name	
wavelet	LLL	first-order	Robust mean absolute deviation	-0.186
original	n/a	shape	Maximum 2D diameter slice	0.113
NIHSS score at baseline				0.105
wavelet	LLL	glcm	Cluster shade	0.040
original	n/a	glszm	Large area low gray level emphasis	0.033
LoG	6 mm	first-order	Skewness	0.023
LoG	6 mm	gldm	Dependence variance	0.011

[a] Feature identifiers are composed of a pre-processing specification (left column: type of pre-processing, i.e. wavelet- or LoG-filtering or original; right column: 3-letter directional specification of wavelet decomposition, or LoG sigma setting), and the feature family and feature name [9, 11].

[b] Regression coefficient from LASSO-LR model. Note that continuous and ordinal variables were standardized before fitting the model.

[c] Only radiomics features included in the radiomics signature were supplied to LASSO-LR models.

CT, computed tomography; GCS, Glasgow Coma Scale; LASSO-LR, least absolute shrinkage and selection operator-regularized logistic regression; LoG, Laplacian of Gaussian; NIHSS, National Institutes of Health Stroke Scale.

Supplemental table 4 Definitions of radiomic features included in signatures.

Feature Family	Feature name	Definition [a]
First-order	Robust Mean Absolute Deviation	"Mean distance of all intensity values from the mean value calculated on the subset of image array with gray levels in between, or equal to the 10th and 90th percentile." [9]
	Skewness	"Measures the asymmetry of the distribution of values about the Mean value. Depending on where the tail is elongated and the mass of the distribution is concentrated, this value can be positive or negative." [9]
Shape	Maximum 2D Diameter Slice	"Largest pairwise Euclidean distance between tumor surface mesh vertices in the row-column (generally the axial) plane." [9]
Texture - Gray Level Cooccurrence Matrix Features (glcm)	Cluster Shade	"Measure of the skewness and uniformity of the GLCM. A higher cluster shade implies greater asymmetry about the mean." [9]
Texture - Gray Level Size Zone Matrix Features (glszm)	Large Area Low Gray Level Emphasis	"Measures the proportion in the image of the joint distribution of larger size zones with lower gray-level values " [9]
	Small Area Emphasis	"Measure of the distribution of small size zones, with a greater value indicative of more smaller size zones and more fine textures." [9]
Texture - Gray Level Dependence Matrix Features (gldm)	Dependence Variance	"Measures the variance in dependence size in the image." [9]

[a] Exact mathematical feature definitions are provided in ref. [9].

Supplemental table 5 Performance of signatures compiled from harmonized radiomic features.

	Discovery cohort [a]		Independent validation cohort
	**Mean CV AUC (SE)**	**AUC (95% CI) [b]**	**AUC (95% CI) [b]**
**Radiomics signature**	0.60 (0.03)	0.66 (0.60 - 0.71)	0.59 (0.54 - 0.65)
**Radiomics + visual signature**	0.60 (0.03)	0.66 (0.60 - 0.71)	0.59 (0.54 - 0.65)
**Radiomics + clinical signature**	0.60 (0.03)	0.66 (0.60 - 0.71)	0.61 (0.55 - 0.67)
**Radiomics + BAT signature**	0.60 (0.03)	0.66 (0.60 - 0.71)	0.59 (0.54 - 0.65)
**Select radiomics [c] + visual signature**	0.65 (0.02)	0.68 (0.62 - 0.73)	0.56 (0.51 - 0.62)
**Select radiomics [c] + clinical signature**	0.65 (0.03)	0.69 (0.63 - 0.75)	0.58 (0.52 - 0.63)
**Radiomics + visual + clinical signature**	0.60 (0.03)	0.66 (0.60 - 0.71)	0.61 (0.55 - 0.67)
**Select radiomics [c] + visual + clinical signature**	0.65 (0.03)	0.69 (0.63 - 0.74)	0.58 (0.52 - 0.63)

[a] The left column shows average test fold AUCs and corresponding SEs across k-fold stratified CV (k = 10, strata: HE-positive and -negative subpopulations) obtained by the "cv.glmnet" R function using optimized lambda parameters; the right column depicts final signatures' performance in the total discovery cohort.
[b] DeLong's method was applied to calculate 95% CIs [22].
[c] Only radiomic features included in the radiomics signature were supplied to LASSO-LR models.

To explore potential performance enhancements, we devised a pipeline iteration incorporating radiomic feature harmonization prior to signature generation to mitigate potential CT slice thickness batch effects, as detailed in the supplemental methods.

AUC, area under the receiver operating characteristic curve; CI, confidence interval; CV, cross validation; LASSO-LR, least absolute shrinkage and selection operator-regularized logistic regression; SE, standard error.

3. Supplemental references

1. Haider SP, Qureshi AI, Jain A, Tharmaseelan H, Berson ER, Zeevi T, et al. Admission computed tomography radiomic signatures outperform hematoma volume in predicting baseline clinical severity and functional outcome in the ATACH-2 trial intracerebral hemorrhage population. European Journal of Neurology. 2021;28(9):2989-3000.
2. Traverso A, Wee L, Dekker A, Gillies R. Repeatability and Reproducibility of Radiomic Features: A Systematic Review. Int J Radiat Oncol Biol Phys. 2018;102(4):1143-58.
3. Haider SP, Burtness B, Yarbrough WG, Payabvash S. Applications of radiomics in precision diagnosis, prognostication and treatment planning of head and neck squamous cell carcinomas. Cancers of the Head & Neck. 2020;5(1):6.
4. Haider SP, Mahajan A, Zeevi T, Baumeister P, Reichel C, Sharaf K, et al. PET/CT radiomics signature of human papilloma virus association in oropharyngeal squamous cell carcinoma. Eur J Nucl Med Mol Imaging. 2020.
5. Haider SP, Zeevi T, Baumeister P, Reichel C, Sharaf K, Forghani R, et al. Potential Added Value of PET/CT Radiomics for Survival Prognostication beyond AJCC 8th Edition Staging in Oropharyngeal Squamous Cell Carcinoma. Cancers (Basel). 2020;12(7).
6. Haider SP, Sharaf K, Zeevi T, Baumeister P, Reichel C, Forghani R, et al. Prediction of post-radiotherapy locoregional progression in HPV-associated oropharyngeal squamous cell carcinoma using machine-learning analysis of baseline PET/CT radiomics. Transl Oncol. 2020;14(1):100906.
7. Zwanenburg A, Leger S, Vallières M, Löck S. Image biomarker standardisation initiative. arXiv e-prints [Internet]. 2016 December 01, 2016. Available from: https://ui.adsabs.harvard.edu/abs/2016arXiv161207003Z.
8. Aerts HJ, Velazquez ER, Leijenaar RT, Parmar C, Grossmann P, Carvalho S, et al. Decoding tumour phenotype by noninvasive imaging using a quantitative radiomics approach. Nat Commun. 2014;5:4006.
9. Pyradiomics-community. Pyradiomics Documentation Release 2.1.2 2018 [cited 2019 December 15th]. Available from: https://readthedocs.org/projects/pyradiomics/downloads/pdf/2.1.2/.
10. Davnall F, Yip CS, Ljungqvist G, Selmi M, Ng F, Sanghera B, et al. Assessment of tumor heterogeneity: an emerging imaging tool for clinical practice? Insights Imaging. 2012;3(6):573-89.
11. van Griethuysen JJM, Fedorov A, Parmar C, Hosny A, Aucoin N, Narayan V, et al. Computational Radiomics System to Decode the Radiographic Phenotype. Cancer Res. 2017;77(21):e104-e7.
12. Friedman J, Hastie T, Tibshirani R. Regularization Paths for Generalized Linear Models via Coordinate Descent. J Stat Softw. 2010;33(1):1-22.
13. Fortin J-P. neuroCombat: Harmonization of multi-site imaging data with ComBat. R package version 1.0.13 ed. github 2023.
14. Orlhac F, Eertink JJ, Cottereau AS, Zijlstra JM, Thieblemont C, Meignan M, et al. A Guide to ComBat Harmonization of Imaging Biomarkers in Multicenter Studies. J Nucl Med. 2022;63(2):172-9.
15. Pszczolkowski S, Manzano-Patrón JP, Law ZK, Krishnan K, Ali A, Bath PM, et al. Quantitative CT radiomics-based models for prediction of haematoma expansion and poor functional outcome in primary intracerebral haemorrhage. Eur Radiol. 2021.
16. McGraw KO, Wong SP. Forming inferences about some intraclass correlation coefficients. Psychological Methods. 1996;1(1):30-46.
17. Koo TK, Li MY. A Guideline of Selecting and Reporting Intraclass Correlation Coefficients for Reliability Research. J Chiropr Med. 2016;15(2):155-63.
18. Revelle W. psych: Procedures for Psychological, Psychometric, and Personality Research. Version 1.8.12 ed. Northwestern University, Evanston, Illinois, USA2018.

19. R Development Core Team. R: A language and environment for statistical computing. Vienna, Austria: R Foundation for Statistical Computing; 2019.
20. Kuhn M, Contributions from Wing J, Weston S, Williams A, Keefer C, Engelhardt A, et al. caret: Classification and Regression Training. 6.0-84 ed2019.
21. Morotti A, Boulouis G, Dowlatshahi D, Li Q, Barras CD, Delcourt C, et al. Standards for Detecting, Interpreting, and Reporting Noncontrast Computed Tomographic Markers of Intracerebral Hemorrhage Expansion. Ann Neurol. 2019;86(4):480-92.
22. DeLong ER, DeLong DM, Clarke-Pearson DL. Comparing the areas under two or more correlated receiver operating characteristic curves: a nonparametric approach. Biometrics. 1988;44:837–45.
